# eGPS 1.0: comprehensive software for multi-omic and evolutionary analyses

**DOI:** 10.1093/nsr/nwz079

**Published:** 2019-06-18

**Authors:** Dalang Yu, Lili Dong, Fangqi Yan, Hailong Mu, Bixia Tang, Xiao Yang, Tao Zeng, Qing Zhou, Feng Gao, Zhonghuang Wang, Ziqian Hao, Hongen Kang, Yi Zheng, Hongwei Huang, Yuzhang Wei, Wei Pan, Yaochen Xu, Junwei Zhu, Shilei Zhao, Ciran Wang, Pengyu Wang, Long Dai, Mushan Li, Li Lan, Yiwei Wang, Hua Chen, Yi-Xue Li, Yun-Xin Fu, Zhen Shao, Yiming Bao, Fangqing Zhao, Luo-Nan Chen, Guo-Qing Zhang, Wenming Zhao, Haipeng Li

**Affiliations:** 1 CAS Key Laboratory of Computational Biology, CAS-MPG Partner Institute for Computational Biology, Shanghai Institute of Nutrition and Health, Shanghai Institutes for Biological Sciences, University of Chinese Academy of Sciences, Chinese Academy of Sciences, China; 2 BIG Data Center, Beijing Institute of Genomics, Chinese Academy of Sciences, China; 3 Computational Genomics Lab, Beijing Institutes of Life Science, Chinese Academy of Sciences, China; 4 CAS Laboratory of Genomic and Precision Medicine, Beijing Institute of Genomics, Chinese Academy of Sciences, China; 5 Shanghai Center for Bioinformation Technology, China; 6 Key Laboratory of Systems Biology, Center for Excellence in Molecular Cell Science, Shanghai Institute of Biochemistry and Cell Biology, Chinese Academy of Sciences, China; 7 Department of Biostatistics and Data Science, School of Public Health, University of Texas Health Science Center at Houston, USA; 8 Shanghai Institute of Biochemistry and Cell Biology, Chinese Academy of Sciences, China; 9 Shanghai Research Center for Brain Science and Brain-Inspired Intelligence, China; 10 Center for Excellence in Animal Evolution and Genetics, Chinese Academy of Sciences, China; 11 University of Chinese Academy of Sciences, China

It has become increasingly challenging for researchers to analyze the exponentially expanding amount of multi-omic data. Here, we describe multi-functional software named evolutionary Genotype-Phenotype Systems (eGPS) that enables users to perform comprehensive multi-omic and evolutionary analyses. The eGPS combines the power of cloud computing and the advantage of desktop application, has a user-friendly graphic interface and is highly interactive. Moreover, third-party plug-ins are supported and their developers can take the credit for contributing the plug-ins. This makes it easier for the community to implement new modules and also encourages their sharing. The eGPS not only develops new functions, tools and methods, but also bridges the gap between multi-omic and evolutionary analyses. The eGPS cloud and desktop application are freely available at http://www.egps-software.org/.

## OVERVIEW OF eGPS

As a new and comprehensive piece of software, eGPS contains more than 20 popular methods and 20 data visualization tools that cover genomics, population genetics, evolutionary biology, differential gene expression and network data analyses. Users can consult the eGPS website and the user manual for further information. The new major functions implemented by the eGPS are outlined below.

### The eGPS cloud

**Figure 1.**
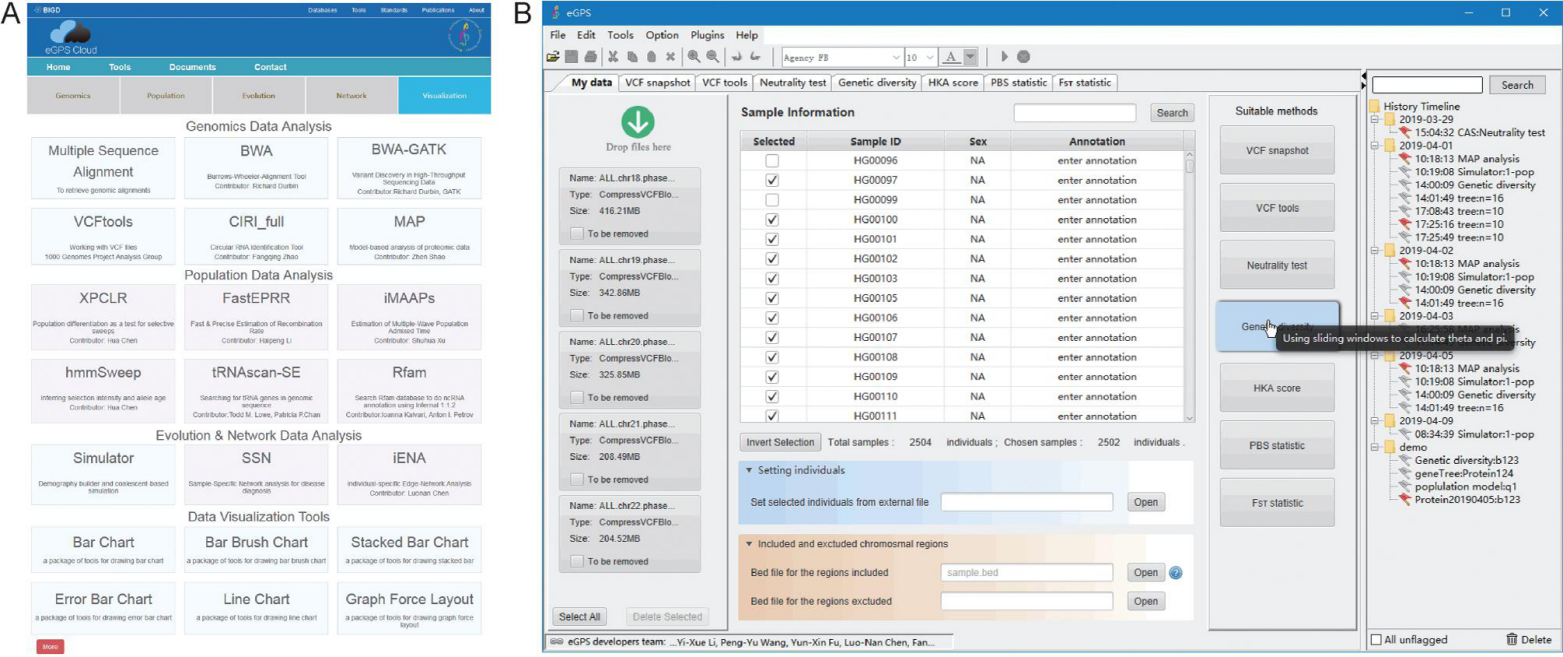
The interfaces of eGPS. (A) The web page of the eGPS cloud, which currently includes 15 software and 20 visualization tools. (B) The eGPS desktop interface consists of a menu, tool bar (top), status bar (bottom), analysis panels and a history panel. The developers of the active analysis panel or (third-party) plug-in are shown in the status bar, while the web page of the developer can be accessed by clicking on the link.

Based on a high-performance computer environment, the eGPS cloud integrates resources for computing and data storage (Fig. 1A). The eGPS cloud is focused on facilitating user-friendly application development (including sophisticated statistical and computational methods) for coherent computational analyses and further promoting the usage of these methods. Tasks are scheduled on high-performance servers and a graphical representation of results will be shown. Meanwhile data protection and privacy are guaranteed by offering secured data processing. Furthermore, the eGPS cloud provides supporting information for the eGPS desktop application when necessary. Currently, there are 480 central processing unit (CPU) cores and >100 TB of high-performance data storage resources in the cloud, which constructs a broad freeway for users to pursue their own scientific interests.

### The eGPS desktop

The user interface of the eGPS desktop has been carefully designed and is easy to use (Fig. 1B). Data files can be easily dragged in and suitable methods will be recommended by the eGPS. When necessary, data files will be indexed for fast access. Individual samples can be included or excluded from further analyses. A searchable history panel is implemented to record the results of analyses obtained by users. History log files can be copied and viewed on another computer where the eGPS desktop application is installed. To compare the results, every analysis panel can be dragged out of the mainframe and become a floating window.

### The Representational State Transfer service for the retrieval of genomic alignments

It is essential for users to obtain sequence alignments when studying the evolution of a gene or a genomic region. The Representational State Transfer (REST) interface is used to retrieve a region of multi-genome alignments, based on a genomic region of the user-defined query genome. The Ensembl REST application programming interface (API) [1] is followed to introduce the required parameters into Uniform Resource Locator and return alignments in the JavaScript Object Notation format.

To avoid loading extra alignments into memory, indexing of multi-genome alignments is an efficient approach [2]. Thus, to efficiently retrieve genomic alignments, an algorithm has been developed to index multi-genome alignments given a query genome. Multi-genome alignments were downloaded from the University of California Santa Cruz genome browser. Indexes were then built according to the coordinates of each genome. Because of gene duplication, a sequence of a non-reference genome can be mapped to different regions of the reference genome. To find its ortholog, we combined neighboring blocks with large segments based on the coordinates of the query genome. Considering the situation of synteny, the longer a reference genome segment is, the more likely it is an ortholog. These index files enable us to achieve fast, random retrieval of alignments given the coordinates of a query genome region. The eGPS currently provides eight sets of species that cover vertebrates, mammals, insects and nematodes. More species sets will be provided, and the eGPS REST APIs will be released publicly in the near future. This REST service is expected to be used widely.

### Gene to gene tree

Multi-omic studies usually provide candidate genes or genomic regions for further evolutionary analyses. The phylogenetic tree is one of the most important concepts in evolution, which helps us to understand the evolution of life and genes, and researchers can also benefit from the phylogenetic tree of a candidate gene. When necessary, the alignment of a gene will be retrieved from the eGPS cloud REST or the Ensembl REST. In the current version, a phylogenetic tree can be constructed by the Swift Neighbor-Joining [3] and other popular tree-building methods, while different genetic distances are provided. The input could be a gene name, a genomic location, an aligned FASTA format file or a pairwise evolutionary genetic distance matrix. A gene tree can also be generated by simply clicking the mouse twice. Users first choose a candidate gene obtained from a multi-omic analysis and then click the ‘build tree’ option from the pop-up menu. This novel function bridges the gap between multi-omic and evolutionary analyses.

### Highly interactive tree viewer

Interactive visualization of a phylogenetic tree is essential in scientific research and education on evolutionary biology, and the visualized tree provides an intuitive sense of evolutionary relationships among species or genes. Hence, a highly interactive graphical visualization tool is implemented for viewing and modifying phylogenetic trees. It has an easy-to-use user interface and many useful functionalities are implemented, such as setting tree layouts, zooming, rotation, clade annotation, display of bootstrap values, the decorating of branch and leaf labels, tree shape modification, un-do/re-do and exporting high-quality figures. Almost all of the operations can be completed by mouse clicking or dragging. In addition, multiple trees can be displayed in different floating windows for comparison with each other.

### Building demographic models and simulating samples like playing Lego

It is essential to generate samples under a variety of demographic models in evolutionary analyses [4]; however, it may be difficult for researchers to implement such models. Thus, a highly interactive tool named ‘simulator’ is implemented. By using a drag-and-drop function, varying demographic models can be constructed as easy as building with blocks. Samples, including ancestral ones, can then be generated by coalescent-based simulation [5,6]. During and after simulation, four summary statistics—namely Watterson's $\theta $, the mean number of nucleotide differences between two sequences, Fu and Li's *D* [7], and Tajima's *D* [8]—will be calculated and their empirical distributions presented. Using floating windows, simulated results under different conditions can be easily compared.

### Third-party plug-in development

An eGPS plug-in is an eGPS component that adds specific features and functions to the eGPS desktop application. It can be developed by third parties, with the developers taking the credit for contributing the plug-ins. It is very easy to install a plug-in, which will be automatically loaded when necessary. Plug-ins can access the eGPS API, thus this feature may cultivate a lively eGPS community. In the current version, two eGPS plug-ins are provided. One is the file format convertor and another is the Fst calculator.

### Network data analyses

A novel tool for performing Sample-Specific Network (SSN) analysis [9,10] is implemented. SSN can detect network biomarkers for disease diagnosis, enabling comparison with traditional molecular biomarkers.

### Pipeline from sequencing reads to visualization of Variant Call Format

The eGPS provides a number of useful pipelines. For example, after quality control, single nucleotide polymorphisms can be called from population sequencing data in the eGPS cloud and results, in Variant Call Format (VCF), can be further analyzed and visualized in the eGPS cloud and desktop application. In particular, VCF is widely used in many genomic applications, but it is not easy to take a glance at VCF files and manipulate them on a personal computer. Thus, VCF tools and a snapshot tool for VCF files are implemented in the eGPS desktop application. Moreover, different summary statistics for genetic diversity and positive selection can be calculated by using sliding windows.

## CONCLUSION

Clouds and desktop applications have their own specific advantages, but few biology software packages can combine both. Therefore, the eGPS cloud and desktop application have been developed combination to address the different needs of users. The eGPS cloud provides easy and free access to a high-performance computing environment, while the eGPS desktop application provides a highly interactive user interface and avoids data transfer. In the next versions, grid computing will be supported in the eGPS desktop application, thus enabling more computing resources to be used for demanding multi-omic and evolutionary analyses. Moreover, the IBM Aspera solution, a proven standard for the high-speed movement of large files, will be supported to optimize data uploading in the eGPS cloud. The Chinese Academy of Sciences Key Laboratory of Computational Biology and BIG Data Center is responsible for the long-term maintenance and updating of the eGPS software and computing resources. Therefore, it is expected that the eGPS will promote the application of multi-omic and evolutionary analyses in more and more studies.

## References

[1] Yates A , Beal K and Keenan S et al. Bioinformatics 2015; 31: 143–5. 25236461 10.1093/bioinformatics/btu613 PMC4271150

[2] Li H , Handsaker B and Wysoker A et al. Bioinformatics 2009; 25: 2078–9. 19505943 10.1093/bioinformatics/btp352 PMC2723002

[3] Saitou N . Introduction to Evolutionary Genomics. London: Springer, 2018.

[4] Xu S , He Z and Zhang Z et al. Natl Sci Rev 2017; 4: 721–34. 31258950 10.1093/nsr/nwx065 PMC6599620

[5] Hudson RR . Gene genealogies and the coalescent process. In: Futuyma D and Antonovics J (eds). Oxford Surveys in Evolutionary Biology Vol 7. New York: Oxford University Press, 1991, 1–44.

[6] Li W-H and Fu Y-X. Coalescent theory and its applications in population genetics. In: Halloran E (ed.). Statistics in Genetics. New York: Springer, 1998.

[7] Fu Y-X and Li W-H. Genetics 1993; 133: 693–709. 8454210 10.1093/genetics/133.3.693 PMC1205353

[8] Tajima F . Genetics 1989; 123: 585–95. 2513255 10.1093/genetics/123.3.585 PMC1203831

[9] Liu X , Wang Y and Ji H et al. Nucleic Acids Res 2016; 44: e164. 27596597 10.1093/nar/gkw772 PMC5159538

[10] Yu X , Zhang J and Sun S et al. Nucleic Acids Res 2017; 45: e170. 28981699 10.1093/nar/gkx787 PMC5714249

